# Predicting growth and curve progression in the individual patient with adolescent idiopathic scoliosis: design of a prospective longitudinal cohort study

**DOI:** 10.1186/1471-2474-11-93

**Published:** 2010-05-17

**Authors:** Iris Busscher, Frits Hein Wapstra, Albert G Veldhuizen

**Affiliations:** 1 University Medical Center Groningen, University of Groningen, Department of Orthopaedics, Hanzeplein 1, 9713 GZ Groningen, The Netherlands

## Abstract

**Background:**

Scoliosis is present in 3-5% of the children in the adolescent age group, with a higher incidence in females. Treatment of adolescent idiopathic scoliosis is mainly dependent on the progression of the scoliotic curve. There is a close relationship between curve progression and rapid (spinal) growth of the patient during puberty. However, until present time no conclusive method was found for predicting the timing and magnitude of the pubertal growth spurt in total body height, or the curve progression of the idiopathic scoliosis.

The goal of this study is to determine the predictive value of several maturity indicators that reflect growth or remaining growth potential, in order to predict timing of the peak growth velocity of total body height in the individual patient with adolescent idiopathic scoliosis. Furthermore, different parameters are evaluated for their correlation with curve progression in the individual scoliosis patient.

**Methods/design:**

This prospective, longitudinal cohort study will be incorporated in the usual care of patients with adolescent idiopathic scoliosis. All new patients between 8 and 17 years with adolescent idiopathic scoliosis (Cobb angle >10 degrees) visiting the outpatient clinic of the University Medical Center Groningen are included in this study. Follow up will take place every 6 months. The present study will use a new ultra-low dose X-ray system which can make total body X-rays. Several maturity indicators are evaluated like different body length dimensions, secondary sexual characteristics, skeletal age in hand and wrist, skeletal age in the elbow, the Risser sign, the status of the triradiate cartilage, and EMG ratios of the paraspinal muscle activity.

Correlations of all dimensions will be calculated in relationship to the timing of the pubertal growth spurt, and to the progression of the scoliotic curve. An algorithm will be made for the optimal treatment strategy in the individual patient with adolescent idiopathic scoliosis.

**Discussion:**

This study will determine the value of many maturity indicators and will be useful as well for other clinicians treating children with disorders of growth. Since not all clinicians have access to the presented new 3D X-ray system or have the time to make EMG's, for example, all indicators will be correlated to the timing of the peak growth velocity of total body height and curve progression in idiopathic scoliosis. Therefore each clinician can chose which indicators can be used best in their practice.

**Trial registration number:**

NTR2048

## Background

Scoliosis is present in 3-5% of the children in the adolescent age group, with a higher incidence in females. The cause of scoliosis in this age group is unknown in the majority of cases (80-85%), and it is therefore called adolescent idiopathic scoliosis (AIS).

Successful treatment of AIS remains a complex challenge for the orthopaedic surgeon. Generally, treatment is conservative (observational or brace therapy) until curves progress to more that 45-50 degrees Cobb angle. Scoliotic curves larger than 50 degrees need surgical treatment. Accurate timing of treatment, whether this is conservative or surgical, plays an essential role in the prognosis of the condition, and in prevention of severe complications like the crankshaft phenomenon[1]. The timing of treatment is mainly dependent on the progression of the scoliotic curve. For optimal treatment, it is important to understand if and when the curve will progress. However, exact prediction of curve progression in patients with adolescent idiopathic scoliosis is still impossible since the prognosis of the deformity is different in each individual.

A known relationship exists between growth of the patient and development of the spinal deformity. It is shown that a rapid increase in (spinal) height at the time of the pubertal growth spurt causes an increase in spinal curvature[2-7]. So, progression of AIS is closely related to rapid growth, and it is therefore likely that understanding longitudinal growth of the patient during puberty is the most important dimension in (treatment of) adolescent idiopathic scoliosis. In order to predict curve progression, it is of great interest to predict the timing and magnitude of the peak growth velocity in the individual patient with idiopathic scoliosis.

The peak growth spurt is known to take place between 10 and 14 years in 95% of the girls, and between 12 and 16 years in 95% of the boys[8,9]. This range holds for a large population, but is too wide to make accurate predictions in the individual patient. However, several other maturity indicators are related to the pubertal growth spurt. Maturity is multidimensional and has various components, including chronological age, skeletal age in different areas, the Risser sign, status of the triradiate cartilage, Tanner stage of sexual maturation, age at thelarche and menarche, and several length dimensions like sitting height, subischial leg length, and foot length or shoe size. These indicators of maturity are all signs that reflect growth or remaining growth of the patient and can be useful in predicting timing of the pubertal growth spurt or peak growth velocity of total body height in the individual patient.

Researchers have tried to find different ways to predict curve progression in AIS as well, besides relating progression to the peak growth velocity of height. A promising method was shown by Cheung et al[3,10], who found a relationship between curve progression and altered electromyography ratios of the convex and concave sides of the curve. Enhanced EMG ratios were observed at three levels of the scoliotic curve (lower end vertebra, apex, and upper end vertebra) at the start of progressive periods. At the start of a non-progressive period, such an enhanced EMG ratio was only observed at the level of the apex vertebra[3].

Only few studies have investigated maturity indicators in patients with idiopathic scoliosis[11-14]. Moreover, studies have compared only some of these indicators, and only either in relationship to the peak growth velocity of total body height, or to curve progression, not to both in one study. Results of different studies are contradictory and inconsistent, so therefore a good algorithm for determining treatment options in AIS is still lacking.

The goal of this study is to determine the predictive value of several maturity indicators that reflect growth or remaining growth potential, in order to predict timing of the peak growth velocity of total body height in the individual patient with adolescent idiopathic scoliosis. Furthermore, different parameters are evaluated for their correlation with curve progression in the individual scoliosis patient. An algorithm is made for determining the optimum treatment strategy in the individual patient with adolescent idiopathic scoliosis.

A description of the maturity indicators is presented below.

### Body length dimensions

It is known that different body length dimensions each have their own typical growth pattern influenced by age- and gender-related factors. Research has been done by Welon and Bielicki[15], Cameron et al[16], Dimeglio[17], and Rao et al[18] to the sequences of peak growth velocities of different length measurements of children from 1-18 years. They all confirm the distal-to-proximal growth maturity gradient as described by Tanner[19]. Body parts that are more distal will have their pubertal growth spurt earlier in adolescence. Furthermore, it is known that the sequence of growth spurts of different length measurements is similar in individual children, regardless of being an "early" or "late" maturer[4,20]. Therefore different body length growth velocities can be useful in predicting peak growth velocity of total height.

In order to calculate growth velocities, access is needed to longitudinal growth data of the patient. It is useful when a patient already has some measurements known at the first visit to the outpatient clinic. Usually that happens very rarely. However, as foot length is said to be the body dimension with the earliest pubertal growth spurt[17,18,21-23], patients were asked if they could recall the course of their shoe size. In a pilot study, it was shown that patients and their parents can recall to a high extend when they bought new shoes and what their shoe size was for approximately the last 2 years. This could therefore be a better measurement than foot length. However, this study should investigate the possibility of a recall bias in a larger dataset, as well as the reliability of the provided information.

### Skeletal age dimensions

Any skeletal region with consistent physeal markers is amenable to be a determination of skeletal age. The most commonly used markers of skeletal age in patients with idiopathic scoliosis are the hand, wrist and the ossification of the iliac apophysis (Risser sign).

Unfortunately, there is a fairly wide range of skeletal age compared with pubertal stages and pubertal growth, although the variation is less than with chronological age.

Determination of skeletal age by hand and wrist X rays is usually done by the method of Greulich and Pyle or Tanner and Whitehouse. The latter is more reliable and reproducible but also fairly complex and time consuming. The Tanner Whitehouse classification can predict well if the patient is pre- or post pubertal growth spurt. The Greulich and Pyle method of assessing skeletal age is easier to use but less precise, and the atlas was compiled from radiographs made in the 1930s [24].

The Risser sign (ossification of the iliac apophysis) continues to be an accepted prognostic sign in the evaluation of growth of patients with idiopathic scoliosis. There is, however, considerable and growing controversy[12-14,25-27]. Disadvantage of using the Risser sign is the wide distribution and the fact that it typically appears after de peak growth velocity of height. Risser stage 1 occurs after the growth spurt in 85% of patients, so its predictive value is very low.

Sanders et al[12] found the status of the triradiate cartilage to be more predictive for the timing of the peak growth velocity of total body height, though it is only predictive whether the patient is before or after the pubertal growth spurt. Once the patient had closed triradiate cartilages, he or she is very likely past the pubertal growth spurt. However, the predictive value of open triradiate cartilages is less clear.

A different method for describing skeletal age is the Sauvegrain method, in which skeletal age is determined using a scoring system for four anatomical landmarks of the elbow. At the onset of puberty, the elbow is still largely cartilaginous. Two years later, fusion of the elbow growth centers is complete. This period is critical since it is marked by the pubertal growth spurt. This method was found to be more accurate and detailed than the Gruelich and Pyle atlas[28]. Furthermore, it is easy to use, and it allows the evaluation of skeletal age in six-month intervals, in contrast to one-year intervals in the most commonly used methods of Gruelich and Pyle, and Tanner and Whitehouse.

### Secondary sexual characteristics

Another hallmark of maturation is de development of secondary sexual characteristics, often classified by Tanner. Thelarche, the first stage of secondary breast development, marks the beginning of puberty and usually takes place 1 year before the peak growth velocity of height[4,11,29,30]. Girls typically reach peak growth velocity of height between stage 2 and 3 of breast development and pubic hair development. The pubertal or Tanner stages are good measurements of maturity. However, most orthopaedic surgeons are uncomfortable in evaluating secondary sexual characteristics with their patients, and self assessment of patients is said to be unreliable. Timing of menarche, the first menstrual period, can be used in girls, but most often takes place 1 year after PHV and is therefore always retrospective. It is assumed that the timing of menarche of the child is closely related to the timing of menarche of the mother. When the mother can recall the timing of her menarche, some predictions can be made for the timing of menarche of the child, and therefore for the timing of the pubertal growth spurt. However, it is still unknown how accurate this relationship is.

## Methods/design

### Study design

A prospective, longitudinal cohort study will be conducted at the outpatient clinic of the department of orthopaedic surgery of the University Medical Center Groningen (UMCG).

The collection of the data will be part of the usual follow up for patients with adolescent idiopathic scoliosis. Approval has been requested from the Medical Ethics Committee for this study, but ethical approval was waived by the Committee, since the collection of the data is part of the usual care in our outpatient clinic.

Informed consent is obtained from each patient and his or her parents before data collection.

### Identification and recruitment of study participants

Patients between 8 and 17 years old, visiting the outpatient clinic of the UMCG with adolescent idiopathic scoliosis will be participating in this study. The Cobb angle should be >10 degrees, measured by an experienced observer on an AP X-ray of the total spine.

Patients should not have had previous spinal surgery; there should be no evidence of neuromuscular disease or skeletal dysplasia, and no evident abnormalities of maturation or height. Furthermore, patients with mental incapacitation are excluded since this can influence the reliability of the measurements in the way that these patients most often can not stand still during the height measurements and EMG measurements. Patients are excluded as well when they are participating in a different study simultaneously.

The assessment to include or exclude a patient will be determined by the orthopaedic surgeon. All patients who meet the inclusion criteria will be asked to participate in this study and give their informed consent.

### Study protocol

The study protocol will be incorporated in the usual care of patients visiting the outpatient clinic for follow up of adolescent idiopathic scoliosis. Follow up will take place every six months. Patients will be followed until 18 years of age or until growth of the spine has stopped and skeletal maturity has been reached. Furthermore, patients will be lost to follow up when they receive surgical treatment.

A description of the (follow up) consult is presented below.

### Anamnestic questions

Besides the regular anamnestic questions (general history, medication, family history, general demographics, present complaints, etc), patients are asked for:

• Height of the parents

• Timing of thelarche, menarche, and menarche of the mother

• History of the shoe size

### Physical examinations

Regular physical exam of the back is done including inspection, determination of the range of motion, and the Adam's forward bending test.

Measurements are taken for total height, sitting height, lateral arm span, foot length, shoe size, and weight. Subischial leg length is calculated by distracting sitting height from total height. All length measurements are measured three times per visit, to the nearest mm, and an average will be calculated.

Measurement of total height is done by use of a stadiometer constructed according to the internationally agreed assignments and with digital reading. The patient is asked to stand upright with bare feet, having their feet parallel together, and the back of the head touching the upright steel rod against the wall. The child is encouraged to stretch out to the maximum by applying gentle pressure upward under the mastoid process, with the recorder watching to see that the heels are not off the ground. The recorder measures height by lowering the horizontal bar of the stadiometer, making contact with the top of the head. No height corrections are made for curve magnitude.

Sitting height is measured with similar height measuring equipment, while the patient sits on a table with exact known height. The patient is asked to sit straight and the legs hanging freely from the ground. Furthermore, measurement of sitting height is similar to the measurement of total height.

Lateral arm span is measured using a tape measure, from the end of the long finger of one hand, to the end of the long finger on the other hand, with arms maximally outstretched. The tape measure is confirmed to the wall, and the patient is asked to stand straight against the wall and stretch out the arms horizontally.

Foot length and shoe size are measured with a validated calliper in which length in mm and standardized shoe size can be determined. Each patient's bare left and right foot are measured individually while standing full weight bearing. The patient stands in the calliper with the back of the heel against the stationary arm, and the movable arm is brought into contact with the tip of the longest toe. The length is measured to the nearest mm, and shoe size to the nearest half-size.

Weight is measured on a validated scale to the nearest 0.1 kg.

Secondary sexual characteristics are determined by the Tanner criteria for breast development and pubic hair development. Determination of the Tanner stage is performed by a trained female observer. Furthermore, patients are asked for self evaluation of the secondary sexual characteristics according to a figure of the Tanner stages. Patients and their parents point out the figure of which they think is most representative. The reliability of self evaluation will be determined by calculating the correlation between the stages of the patient and the trained observer.

### X-ray examinations

Since a clinically important increase in curve severity exists between morning and evening [31], all X-ray examinations are taken in the morning using a standardized X-ray protocol. Brace treated patients are required to remove their brace the morning of the X-ray examination.

All X-ray examinations are performed with a new ultra low dose 2D/3D digital X-ray system (Biospace Med, Paris, France).

**Figure 1:**
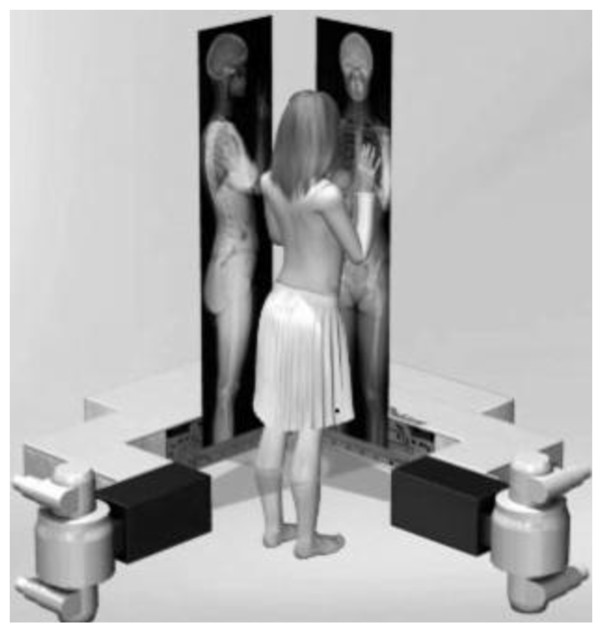
**Images obtained by the ultra-low dose 2D/3D digital X-ray system used in this study**. The system can provide total body X-rays for AP and lateral planes simultaneously

This system allows for the simultaneous acquisition of two orthogonal planar images in a vertical scanning mode. The gantry is composed of two sets of detectors and X-ray tubes positioned orthogonally and supported by a mobile C-arm. This C-arm moves vertically using a linear scanning technique. The patient is positioned at the intersection of the two X-ray beams which scan the patient vertically, as illustrated in Figure 1. A single scan will simultaneously produce both AP and lateral images of the patient. A huge advantage of making a total body X-ray is that multiple measurements can be performed in just one single X-ray.

**Figure 2:**
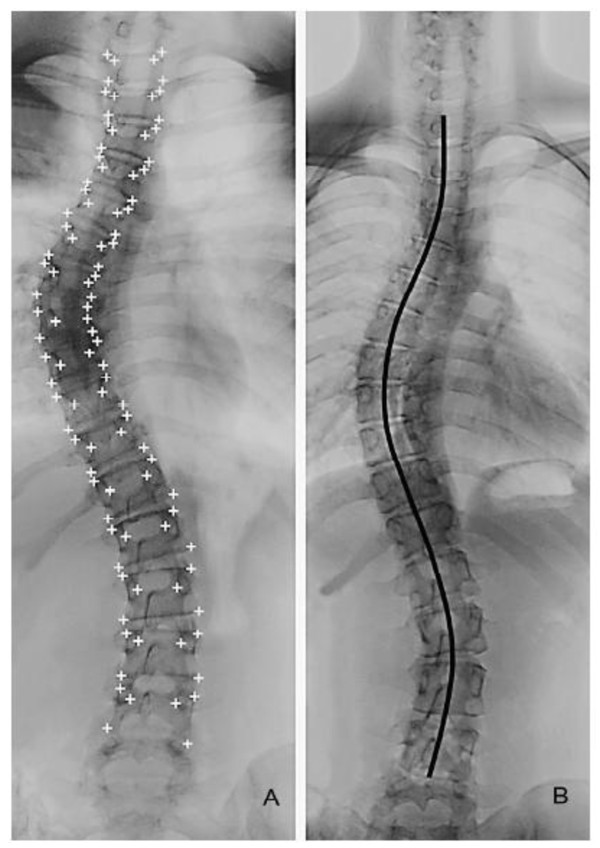
**Calculation of the length of the scoliotic spine as described by Cheung et al.** The length is measured from T1 to L5 through the midpoints of all vertebrae and discs.

On the AP X-ray the scoliosis is classified and the Cobb angle is measured according to internationally agreed standards. The length of the scoliotic spine is measured from the upper end-plate of T1 to the lower end-plate of L5 (Figure 2), according to a previously described method by Cheung et al[32]. Furthermore the axial rotation and lateral deviation of each vertebra are determined.

Determination of the state of the triradiate cartilage is performed on the AP X-ray of the pelvis (open or closed), as well as determination of the Risser stage (stage 1-5).

Skeletal age of the hand and wrist is determined by a trained observer according to the method of Tanner and Whitehouse. Skeletal age of the elbow is assessed by the Sauvegrain method, using a scoring system for four anatomical landmarks of the elbow[28].

### Complementary examinations

An electromyography is made of the paraspinal muscles in order to determine the ratios of muscle activity on the convex and concave side of the main scoliotic curve.

**Figure 3:**
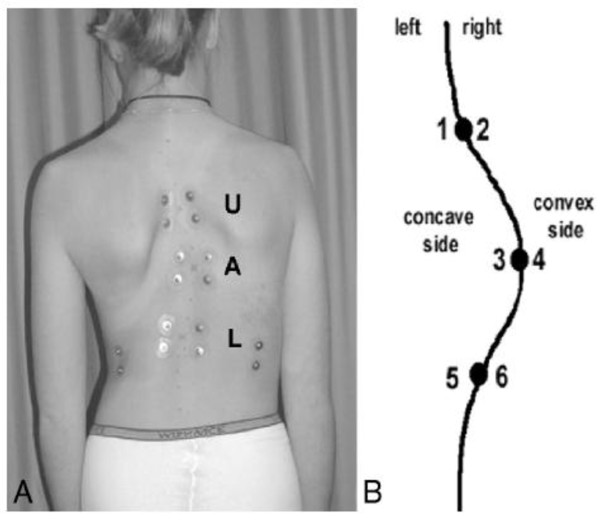
**The placement of 6 pairs of bipolar surface EMG electrodes on the paraspinal muscles of the scoliotic spine**. L indicates the lower end-vertebra, U the upper end-vertebra, and A the apex vertebra

For the electromyographic measurements, 12 EMG electrodes are placed symmetrically along the superficial erector spinae muscles at three levels, 30 mm from the midline, and parallel to the spinous processes (figure 3). The electrode levels correspond to both end vertebrae of the curve (most tilted ones), and the apex vertebra (Figure 3).

The ECG is measured from two electrodes at the sternum and ictus cordis. The electrodes are connected to a multichannel recording device (Porti system, TMS International, Enschede, The Netherlands). Raw EMG signals are amplified, AD-converted, and stored at a sampling rate of 800 Hz in a computer for analysis. The EMG signals are full-wave rectified and low-pass filtered. Reliability data for these specific EMG measurements are available from Cheung et al [3].

The EMG signals are recorded in 5 postures: 1) with the patient in a relaxed upright standing posture with the arms along the body and feet together, 2) with the patient bending to the right, 3) with the patient bending to the left, 4) with the patient bending forward, 5) with the patient sitting in a relaxed position in a chair, with the arms along the body and the back against the chair.

The paraspinal activity (EMG) ratio is defined as the EMG activity of the erector spinae muscles between a convex electrode pair divided by the EMG activity between a contralateral concave electrode pair. The EMG ratios of all 3 levels are considered.

### Outcome measurements

Longitudinal data will be acquired for:

• Scoliotic curve progression (in degrees Cobb angle)

• Height, and growth velocity of height

• Sitting height, and growth velocity of sitting height

• Leg length, and growth velocity of leg length

• Foot length, shoe size, and growth velocity of foot length and shoe size

• Weight, and growth velocity of weight

• Spinal length, and growth velocity of spinal length

• Tanner stage of secondary sexual characteristics

• Riser sign and status of the triradiate cartilage

• Skeletal age in hand, wrist, and elbow

• EMG ratios of the paraspinal muscles

**Figure 4:**
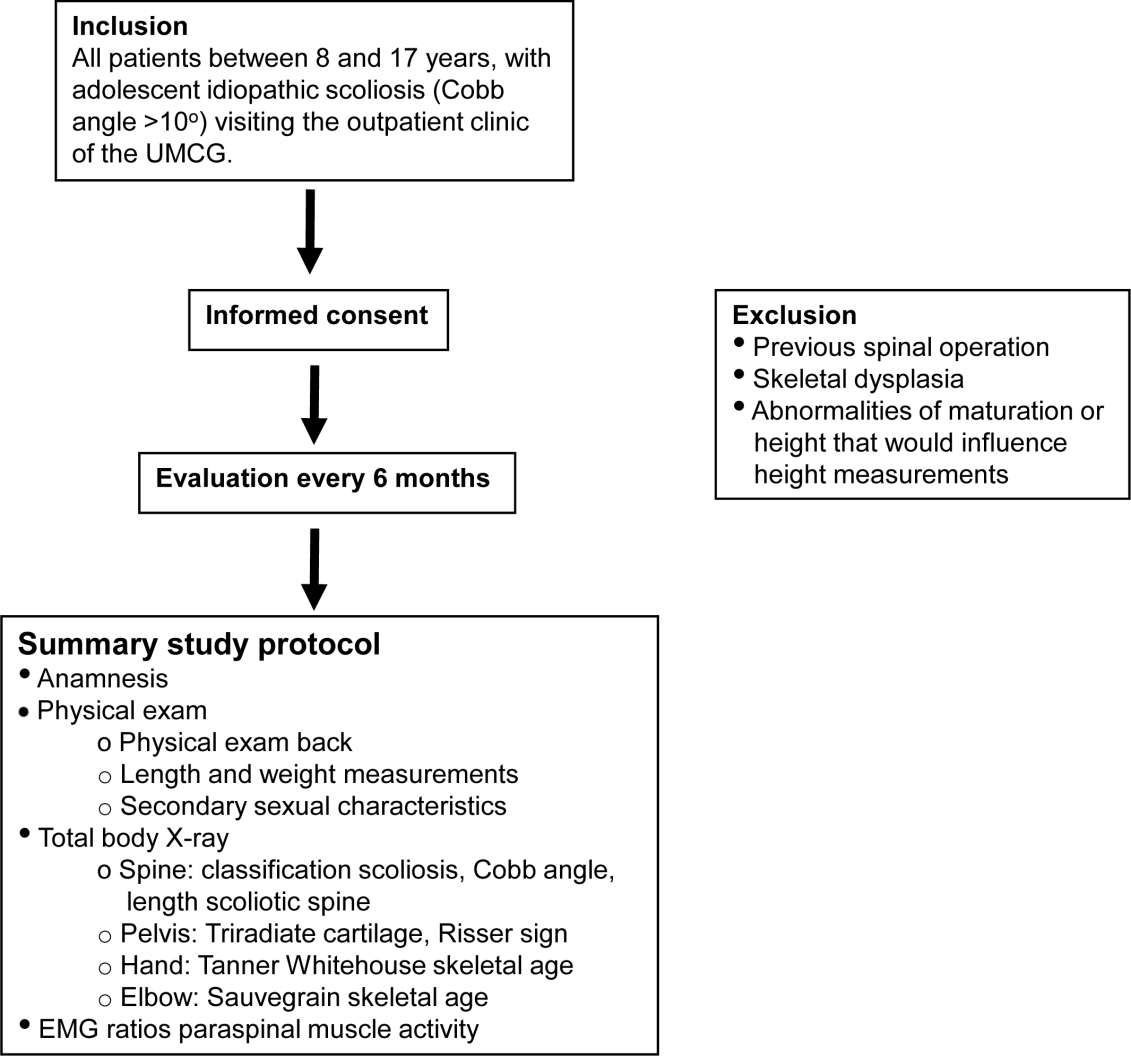
**Summary of the study protocol**.

Finally, an algorithm will be developed for predicting peak growth velocity of height and curve progression in individual patients with adolescent idiopathic scoliosis (see Figure 4). 

### Time frame

The first patients will be included in January 2010. An exact date for ending of this study is not provided as it will be incorporated in the usual care of AIS patients, and it is aimed to proceed for the coming years. The first evaluation of the results is expected to take place 2.5 years after the start of the study, in July 2012. In that time, 25 patients are expected to have fulfilled a two-year follow up time.

### Sample size and statistical analysis

The maximum sample size of this study is not determined on beforehand, as the study is aimed to proceed for the coming years.

We aim to perform a first preliminary analysis of the longitudinal data when patients have a follow up of at least 2 years. As the expected number of new patients is 60 each year, it will take 2.5 years for 30 patients to reach a follow up of at least 2 years. Taking a loss to follow up into account due to surgical treatment, we expect to be able to analyse the complete data of at least 25 patients. The statistical power will increase with a longer follow up and more patients. The number of 25 patients was checked whether it was large enough to obtain a good prediction model according to the criteria of Knofczynski and Mundfrom [33]. With a significant large influence of 4-5 predictor variables (as is expected in this study) and a squared multiple correlation coefficient between 0.5 and 0.7 between 25 and 65 patients should be included. Therefore the first preliminary analysis will be performed using data of 25 patients.

Individual data charts are made, and results of each parameter are plotted against the growth velocity of total body height and the curve progression.

**Figure 5:**
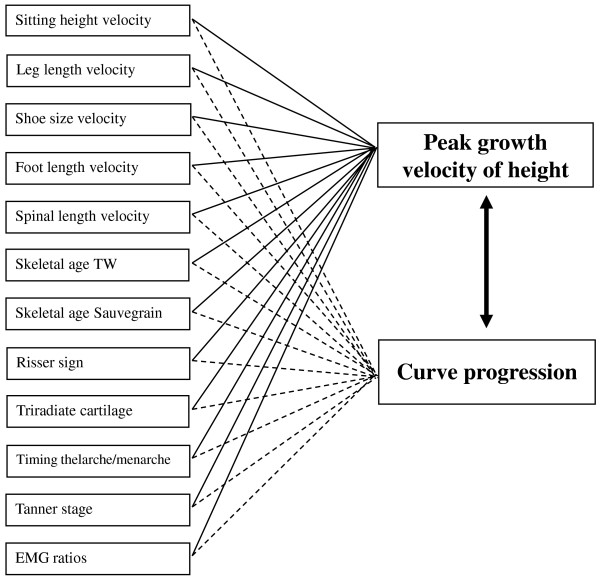
**Correlations investigated in this study**.

All statistical calculations are made using SPSS (SPSS Inc, Chicago USA). Pearson correlation coefficients are computed for each individual possible related parameter against the peak growth velocity and the curve progression (Figure 5). A correlation above 0.70 is believed to be clinically relevant. To be able to interpret this value in a reliable way, we will report the confidence intervals as well.

Multiple linear regression analysis of the data is used to determine predictive equations for the timing and magnitude of the peak growth velocity of height, and the scoliotic curve progression.

Missing data concerning individual parameters are not expected in this study. I.e. when a patient misses his or her follow up consult, *all *parameters will be missing and therefore no correlations can be calculated for that time point. It is not expected that when a patient does come to the outpatient clinic, for example the sitting height will not be measured and all the other parameters will be measured. If it does happen anyway that one individual parameter is missing, imputation techniques will be applied during data analysis.

## Discussion

Many researchers are searching for an accurate method to predict curve progression in adolescent idiopathic scoliosis, and related to that, timing and magnitude of peak growth velocity of height. Until this time, no conclusive methods were found, as many researchers could not determine all maturity indicators in one study, and results of different studies were contradictory. A major advantage of present study is the use of a new ultra low dose 2D/3D digital X-ray system. Therefore it is possible to determine skeletal age dimensions in many regions with just a single, total body X-ray. Furthermore, to our knowledge this is the most complete study for evaluation of maturity indicators, including an extensive anamnestic evaluation, measurement of various body length dimensions, evaluation of secondary sexual characteristics, and an extensive radiological assessment. Furthermore, an extra predictor of curve progression will be evaluated by measuring EMG ratios of the paraspinal muscles.

Blood serology of different growth factors or hormones will not be determined in this study since most parameters are only conclusive retrospective, and the variation of values is too large to be useful. Furthermore, values of many hormones are variable during the day, and as is it logistically very difficult to measure each patient at a precise similar moment, this would influence the results too much. The last reason for not including blood serology is the burden on the adolescent patient by this invasive test.

Purpose of this study is to acquire a large database of longitudinal maturity indicators of patients with adolescent idiopathic scoliosis. This database should result in a reliable and reproducible algorithm for decision making in treatment of AIS. However, this database will also provide a tool for many physicians involved in paediatric medicine in general. In a vast amount of paediatric disorders it is of importance to understand when the peak growth velocity takes place, particularly when a proposed therapy or surgery may affect the growth and ultimate length. Examples are deviated growth in children needing growth hormone therapy, idiopathic precocious puberty needing GnRH analog therapy, and leg length differences needing growth arrest in the physis or contrary, leg lengthening[34]. This database can help in developing treatment strategies in many disorders.

As mentioned before, the use of a new ultra low dose X-ray system is a huge advantage of this study. However, not all clinicians will have access to a system like that, and can not determine skeletal age in many different regions, as radiation exposure will be too high for the patient. Furthermore, not all clinicians will have the time and space to perform many measurements in their outpatient clinic. Therefore correlations with peak growth velocity of total body height and curve progression are calculated for each individual parameter as well, so that researchers and clinicians can choose which parameter is interesting and feasible in their practice.

In conclusion, this study will provide an extensive and valuable database of many (growth) parameters related to the peak growth velocity of total body height and the curve progression in patients with adolescent idiopathic scoliosis. This information will be helpful in determining the optimum treatment strategy for patients with AIS and therefore in prevention of complications and improvement of the prognosis.

## Competing interests

The authors declare that they have no competing interests.

## Authors' contributions

All authors have read and approved the manuscript and believe the manuscript represents honest work. All authors (IB, FHW, and AGV) have made substantial contributions to the design of the presented study. IB has written the first manuscript. FHW and AGV have performed the reviewing of the manuscript.

## Pre-publication history

The pre-publication history for this paper can be accessed here:

http://www.biomedcentral.com/1471-2474/11/93/prepub
